# Distribution and Probabilistic Risk Assessment of Antibiotics, Illegal Drugs, and Toxic Elements in Gastropods from Southeast China

**DOI:** 10.3390/foods13081166

**Published:** 2024-04-11

**Authors:** Hai-Tao Shen, Xiao-Dong Pan, Jian-Long Han

**Affiliations:** Zhejiang Provincial Center for Disease Control and Prevention, Hangzhou 310051, China

**Keywords:** antibiotics, gastropod, heavy metals, leucomalachite green, risk assessment

## Abstract

We investigated fourteen antibiotics, three illegal drugs, and two toxic elements in commercially available gastropods from southeast China. The data revealed high detection frequencies (DFs) for florfenicol (61.32%), florfenicol amine (47.33%), and thiamphenicol (39.88%), with maximum concentrations of 1110, 2222, and 136 μg/kg wet weight (ww), respectively. The DFs of illegal drugs were 3.54% for leucomalachite green and 0.3% for chloramphenicol. The average levels of Cd and As were 1.17 and 6.12 mg/kg ww, respectively. All chemicals presented diverse DFs in different sampling months. The highest DFs of florfenicol, florfenicol amine, and thiamphenicol were in July. The health risk assessment showed that targeted hazard quotients (THQs) of antibiotics, Cd, and As for children, teens, and adults were all less than one. Notably, the toxic elements (Cd and As) were identified as the primary health risk in gastropods, contributing to over 90% of the total THQs.

## 1. Introduction

Mollusks, including gastropods and bivalves, are popular dietary products in many regions, particularly in coastal cities. China is a potential global leader in seafood production, notably in mollusk aquaculture. According to the Chinese Fisheries Statistical Yearbook 2021, farmed marine gastropod production in the southeastern provinces of Zhejiang and Fujian reached 0.6 million tons in 2020 [1].

Gastropods, valued for their nutrition and unique flavors, play a significant role in the local seafood industry. However, rapid industrial development has led to marine pollution, and high-density farming practices have increased the use of animal drugs or illegal chemicals, resulting in residues in seafood [2,3]. To ensure the safety and sustainability of the marine aquaculture industry, it is essential to address the potential presence of contaminants, including chemical drugs, and toxic elements.

The global concern over antibiotic-resistant bacteria has heightened due to the presence of antibiotics in food products. Excessive and improper antibiotic use in both human and veterinary medicine has contaminated various food sources, including seafood. Li et al. (2012) identified widespread antibiotic residues in mollusks from the Bohai Sea, northeast China, primarily quinolones, ranging from 0.71 to 1575.10 μg/kg [4]. Chiesa et al. (2018) reported oxytetracycline in mollusks from the North Adriatic Sea, Italy, averaging 125 μg/kg [5]. The presence of antibiotics in seafood poses a significant health risk, contributing to antibiotic resistance development and adverse effects on the human body.

In addition to antibiotics, the illegal use of drugs in aquaculture, including veterinary drugs and growth promoters, has raised serious concerns. For instance, our previous report discovered steroid hormone residues in farmed fish [6]. Malachite green (MG), initially used for treating parasitic, fungal, and protozoan diseases in aquaculture, is legally restricted or prohibited in many countries due to its toxic effects on humans, including potential carcinogenic properties and risks to the liver and kidney [7]. Hashimoto et al. (2011) documented the presence of MG and its metabolite, leucomalachite green (LMG), in farmed fish from Brazil [7]. These chemical residues in food have detrimental effects on human health. Moreover, the illegal use of drugs in aquaculture can lead to environmental pollution and disrupt the delicate balance of ecosystems.

Shellfish, particularly gastropods, are known for accumulating toxic elements, such as Cd, As, and Hg, from their surrounding environment. These elements can enter the food chain through various pathways and eventually accumulate in gastropods [8]. Our previous study found high Cd content, averaging 0.581 mg/kg, in mollusks from Zhejiang, southeast China [9]. Consumption of gastropods contaminated with toxic elements can result in adverse health effects, including organ damage and developmental issues in vulnerable populations such as children and pregnant women.

It is important to assess the chemical contaminant occurrence in aquatic products, considering potential risks associated with dietary exposure. However, to the best of our knowledge, there are few reports about contaminant distribution in gastropods from southeast China and health risk assessment [9,10,11]. This study aims to investigate antibiotics, illegal drugs, and toxic elements in gastropods marketed in southeast China and assess dietary exposure to the local population. These data can assist in establishing appropriate regulations and guidelines to ensure food safety and protect public health.

## 2. Material and Methods

### 2.1. Reagents and Chemicals

Chemical standards for ciprofloxacin, enrofloxacin, lomefloxacin, norfloxacin, norfloxacin-d_5_, ofloxacin, pefloxacin, chlortetracycline, doxycycline, oxytetracycline, tetracycline, tetracycline-d6, chloramphenicol, chloramphenicol-d_5_, thiamphenicol, florfenicol, florfenicol amine, metronidazole, metronidazole-d_4_, malachite green, malachite green-d_5_, Leucomalachite green, and leucomalachite green-d_5_ were all purchased from Sigma-Aldrich with purity grade more than 95% (St. Louis, MO, USA). The standard solutions of arsenic (As) and cadmium (Cd) were brought from Agilent Technologies (Santa Clara, CA, USA). HPLC-grade acetonitrile (ACN), methanol (MeOH), formic acid (FA), and ethyl acetate were brought from Merck Chemicals Ltd. (Darmstadt, Germany). Ultrapure HNO_3_ (68.0–70.0%, Certified ACS) was obtained from Fisher Scientific (Ottawa, ON, Canada). Ammonia solution, hydrochloric acid, sodium chloride, and ethylenediaminetetraacetic acid disodium salt (Na_2_EDTA) with analytical grade were all purchased from Sinopharm (Beijing, China). Ultrapure water was produced through the Milli-Q ultrapure system (Millipore, Bedford, MA, USA).

### 2.2. Sample Collection

**Figure 1:**
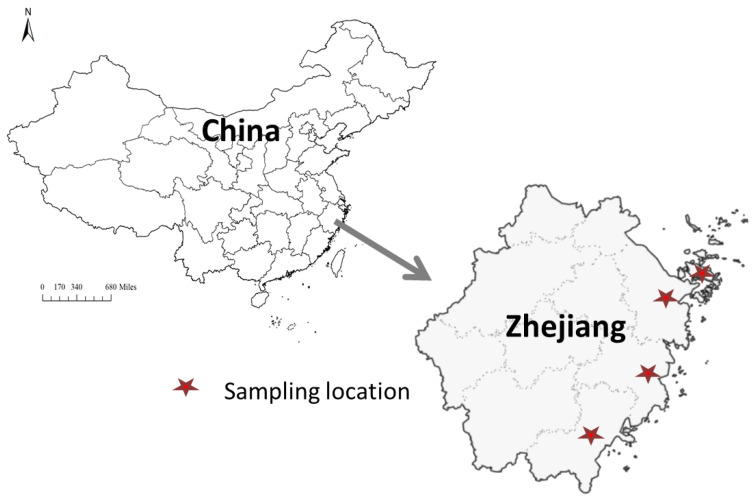
The simple map of sampling areas from Zhejiang, southeast China.

A total of 637 marine gastropod samples were randomly collected from Zhejiang marine areas, in southeast China, over three consecutive years (2020–2022). The sampling regions are illustrated in Figure 1. All gastropods (*Babylonia lutosa*) with edible parts were promptly dissected, homogenized in the laboratory, and stored at −20 °C until instrumental analysis. The storage time for frozen samples did not exceed 7 days before analysis. 

### 2.3. Analysis of Antibiotic Residues and Illegal Drugs 

Antibiotic residues QNs (ciprofloxacin, enrofloxacin, lomefloxacin, norfloxacin, ofloxacin, pefloxacin), TCs (chlortetracycline, doxycycline, oxytetracycline, and tetracycline), CAPs (thiamphenicol, florfenicol, florfenicol amine, and chloramphenicol), NMZ (nitroimidazole), and illegal drugs (malachite green and its metabolite leucomalachite green) were all analyzed using UPLC–MS/MS methods with MRM scan mode in laboratories. 

**Antibiotics.** The analysis methods for quinolones (QNs) and tetracyclines (TCs) were based on a previous method [12]. In summary, 2.0 g of sample was mixed with 20 mL of 0.1 mol/L EDTA-Mcllvaine buffer for analyte extraction. The extraction was purified using an Oasis HLB SPE cartridge (200 mg, 6 mL, Waters, Milford, CT, USA). The cartridge was pre-conditioned with 8 mL of methanol and 8 mL of ultrapure water. After sample loading, the cartridge was washed with a mixture of 5 mL of methanol/water (volume ratio of 5/95), and the analytes were eluted with 8 mL of methanol. The elution was collected, dried under a flow of nitrogen, and reconstituted with 1 mL of 0.1% formic acid solution for instrumental analysis.

Chloramphenicol (CAPs) and nitroimidazole were tested using the following procedures. Briefly, 2.0 g of sample was extracted with 10 mL of ethyl acetate containing 2% ammonium hydroxide. The extraction was then centrifuged at 8000× *g* for 10 min, and the supernatant was collected. The supernatant was purified using an Oasis MCX SPE cartridge (6 cc/500 mg, Waters, USA). The cartridge was pre-conditioned with 6 mL of methanol and 6 mL of ethyl acetate. After sample loading, the cartridge was washed with 5 mL of ethyl acetate and 5 mL of methanol. The analyte was eluted with 6 mL of 5% aqueous ammonia solution, collected, and dried under a flow of nitrogen. It was then reconstituted with 1 mL of 10% methanol solution for instrumental analysis.

**Illegal drugs (malachite green and its metabolite leucomalachite green).** A 5.0 g ground sample was transferred into a plastic centrifuge tube. The analytes were extracted with 25 mL of acetonitrile with a vortex mixer. The samples were centrifuged at 8000× *g*/min for 10 min, and the supernatant was collected and purified with SPE. Neutral alumina SPE (Sep-pak 6 cc/500 mg Waters, USA) was pretreated with 5 mL acetonitrile, and the supernatant was loaded onto the SPE. A 4 mL acetonitrile was added for washing. All elution was collected, and dried by nitrogen flow at 50 °C. The residue was re-diluted with 1 mL 30% ACN for instrumental analysis.

All organic analytes were analyzed using an ultra-high-performance liquid phase coupled with a triple quadrupole mass spectrometer. Chromatographic separation was performed on a chromatographic UPLC BEH C_18_ column (Waters ACQUITY, Milford, CT, USA). The chromatographic column temperature was maintained at 40 °C, and the liquid flow rate was set to 0.4 mL/min. A gradient elution program was employed with specific conditions detailed for different analytes in Supplemental Tables S1 and S2. The mass spectrometry utilized electrospray ionization (ESI) as the ion source, and the analysis employed the MRM channel mode. Detailed mass spectrometry parameters for the target analyte are provided in Supplemental Table S3.

### 2.4. Analysis of Elements 

The determination of elements As, Cd, Hg, and Pb was performed using inductively coupled plasma mass spectrometry (ICP-MS) based on our previous literature report [11]. Briefly, 0.5~1.0 g of sample was weighed and transferred to a polytetrafluoroethylene container. Then, 6 milliliters of nitric acid were added, and the digestion was carried out by a closed-vessel microwave digestion system. The digestion program was set to raise the temperature from room temperature to 195 °C within 20 min and maintained for 20 min. The digestion solution was heated at 150 °C to dryness until the solution was almost dry. Finally, the residue was transferred by 20 milliliters of deionized water into a centrifuge tube for instrumental analysis. The parameters of ICP-MS are shown in Supplemental Table S4. 

### 2.5. Method Validation

To control the analysis quality, the method’s precision and accuracy were validated in a local laboratory. Spiking samples with different standards were conducted through the blank sample matrix for QNs, TCs, CAPs, metronidazole, malachite green, and leucomalachite green. Spiking recovery was used to evaluate the accuracy and the relative standard deviation (RSD) for method precision. Certified reference materials were utilized for elemental analysis to ensure analysis quality. The limit of quantification (LOQ) was calculated as the concentration corresponding to a 10 times signal-to-noise (S/N) ratio.

### 2.6. Probabilistic Risk Assessment

The targeted hazard quotient (THQ) was used to estimate the health risk associated with a single chemical compound, and the hazard index (HI) for the cumulative effect of multiple substances. Considering the similar biological properties of antibiotics, the total effect was estimated by adding up the individual exposures. The formulas are shown as follows:

$$EDI=\frac{C\times IR}{BW}$$

$$THQ=\frac{EDI}{ADI}$$

$$HI=\sum_{i=1}^{n}THQ\;$$

EDI is the estimated daily intake, where C represents the concentration of the compound in gastropods, and IR is the ingestion rate for a day. The daily consumption of aquatic products is 35.92 g for children, 73.44 g for teens, and 103.91 g for adults, based on the report of the Zhejiang provincial Food and Drug Administration, China [13]. It is assumed that gastropod consumption accounts for 20% of the total aquatic products. BW, body weight, is 16.68 kg for children (age 4 to 11), 46.25 kg for teens (age 12 to 17), and 57.03 kg for adults (over 18) [14]. The ADI is the acceptable daily intake based on the US FDA [15] and a published report [16]. HI is obtained by the sum of the individual THQ. When the HI value is more than 1, it indicates a moderate or high risk of harmful effects on human health.

The method for health risk assessment of toxic metal was described in our previous reports [9,10,11,14,17]. Briefly, the targeted hazard quotient (THQ) was employed to estimate non-carcinogenic health risks. It is calculated as follows.

$$THQ=\frac{EDI}{RfD}$$

TR=EDI×CSF 

RfD is the oral reference dose, and the values for inorganic As and Cd, are 0.3 μg/kg bw per day and 1 μg/kg bw per day, respectively. The concentration of inorganic As for As (III) and As (V) accounted for about 10% of the total As in marine products. For carcinogenic health risk, the lifetime target cancer risk (TR) was calculated. CSF is the cancer slope factor, and the value for inorganic As is 1.5 mg kg^−1^ day^−1^. TR of As was calculated based on inorganic As, which accounts for 2% of the total As.

Considering MG and LMG are both genotoxic and carcinogenic substances, their risk assessment was performed with a margin of exposure (MOE) based on a previous report [18]. MOE is calculated as follows.

$$MOE=\frac{BMDL}{EDI}$$

BMDL is the benchmark dose lower confidence limit. BMDL_10_ benchmark value of 13 mg/kg bw per day for neoplastic effect and BMDL_05_ of 6 mg/kg bw per day for non-neoplastic effect were used according to the European Food Safety Authority [19]. 

All calculations were performed using Monte Carlo (MC) simulations, with 10,000 runs executed by Oracle Crystal Ball (version 11.1.34190, free trial version, Oracle Corporation, USA) in Microsoft Excel™ 2010. The parameters of Monte Carlo simulations were listed in Supplemental Table S5 based on our previous report [14]

## 3. Results and Discussion 

### 3.1. Method Validation 

For the method validation of organic chemicals, a blank matrix spiked with different levels of standards was employed. The spiking recoveries and relative standard deviations (RSDs) were calculated to assess the accuracy and precision. A series of isotope-labeled standards were used as the internal standards (Supplemental Table S3). As shown in Table 1, the correlation coefficients (*r*^2^) of linear calibration were all greater than 0.995. The limits of quantification (LOQs) ranged from 0.1 to 3.0 μg/kg. All spiking recoveries were between 80% and 110%, and the RSDs were all less than 15%. 

Regarding toxic elements, we used the certified reference material (GBW10068 Oyster) for validation. The measured values of As and Cd were similar to the certificated values, with the RSDs of the analyzed concentrations less than 10% (Table 2).

### 3.2. The Distribution of Antibiotics, Illegal Drugs, and Toxic Elements

Fourteen antibiotics, three illegal drugs, and two elements were determined in gastropod samples (Table 3). The detection frequency, representing the percentage of detected samples to the total analytical samples, was used to describe the overall presence of contaminants. Two toxic elements (As and Cd) had a higher detection frequency (>95%) than other organic chemicals due to their widespread presence in aquaculture products. The maximum residue limits (MRLs) for As (inorganic As) and Cd are 0.5 mg/kg wet weight (ww) and 2.0 mg/kg ww, respectively, based on the Chinese government [20]. For antibiotics, the MRLs have been recommended by Commission Regulation 37/2010 [21] and the Chinese Agriculture Minister (GB-31650) [22] only for fish, not shellfish.

**Antibiotics.** Antibiotics of diverse types are commonly used in aquaculture production in many countries. It was observed that 11 of the 14 antibiotics were found in gastropod samples from southeast China. Antibiotic residues with high detection frequency were florfenicol (61.32%), florfenicol amine (47.33%), and thiamphenicol (39.88%), with the maximum concentration of 1110, 2222, and 136 μg/kg wet weight, respectively. Ciprofloxacin and oxytetracycline had detection frequencies of 6.21% and 6.02% with maximum concentrations of 1110 and 3410 μg/kg ww, respectively. 

The use of antibiotics in aquaculture is primarily for disease prevention and treatment, but overuse and misuse can lead to the development of antibiotic-resistant bacteria in aquatic environments [23], posing serious implications for human health. QNs, especially enrofloxacin and its metabolite ciprofloxacin are usually detected in marine fish, as reported by previous studies [24,25,26,27]. However, our study revealed that antibiotics with high detection frequency were CAPs and TCs in gastropod samples, unlike fish. Similar results were found in clams from the North Adriatic Sea, Italy, which contained tetracycline (49.45 μg/kg ww), oxytetracycline (125.03 μg/kg ww), doxycycline (60.45 μg/kg ww), and chlortetracycline (77.48 μg/kg ww) [5]. Chloramphenicol has been prohibited for its usage in aquaculture due to its serious toxicity, so it was also classified into groups of illegal drugs. We found chloramphenicol with a detection frequency of 0.3% in gastropods. A high detection frequency (52%) of chloramphenicol was found in clams (*Ruditapes philippinarum*) with a mean level of 66 μg/kg ww from Guangdong, south China [28]. 

**Illegal drugs**. Malachite green (MG), and Leucomalachite green (LMG) are prohibited for use in aquaculture by many countries due to their severe health risks [18,29]. In this study, MG was not detected in any gastropod samples. The detection frequencies of chloramphenicol and LMG were all less than 5%. Previous reports have focused on MG and LMG residues in farmed fish rather than in shellfish [7,30,31]. Despite the regulations, illegal use of these substances continues in some parts of the world, driven by the need to control diseases in aquaculture and the lack of effective alternatives [2]. This has led to ongoing efforts to improve surveillance and enforcement, as well as to develop safe alternatives to these harmful substances. 

**Toxic elements.** Bivalves and gastropods are usually selected as bio-indicators of marine environmental pollution [32]. Our data showed that the average levels of Cd and As were 1.17 and 6.12 mg/kg ww. The highest level for Cd was 20.8 mg/kg ww, and for As, it was 48.2 mg/kg ww. Gouveia et al. revealed a high mean As concentration (36.8 mg/kg ww) and a low Cd level (less than 1 mg/kg ww) in gastropod tissues from Brazil [33]. Barchiesi et al. found the Cd in gastropod samples from Italy to be 0.12~0.30 mg/kg ww, which was lower than our result [34]. A high mean Cd concentration of 5.33 mg/kg ww was observed in gastropod (*M. trapa*) from south India [35]. Another report showed the average level of Cd in gastropod (*P. globosa*) from south India was 0.28 mg/kg ww [36]. The combination of environmental pollution, biological species, and ecological factors can contribute to the accumulation of toxic elements in gastropods. Monitoring these factors is important for understanding the potential impacts of toxic element contamination on gastropods.

### 3.3. The Effect of Sampling Data

**Figure 2:**
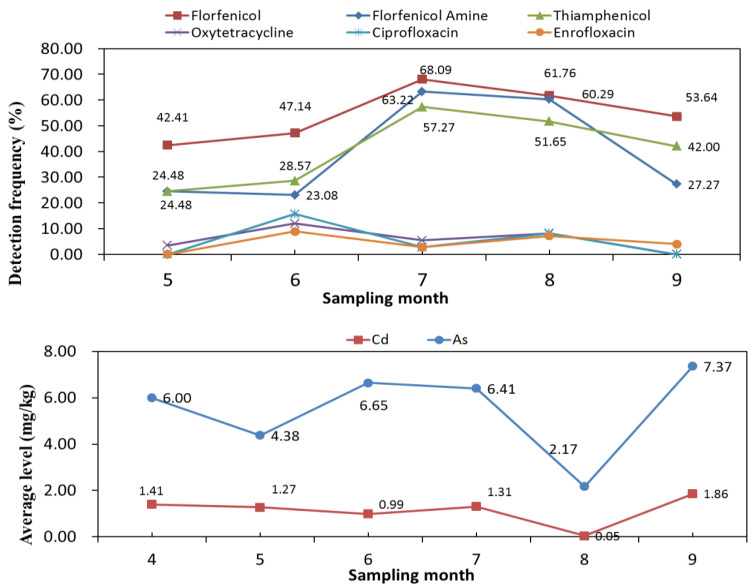
The detection frequency of antibiotics and the mean concentration of elements in gastropods collected in different months.

We investigated the effect of sample collection data on the concentration of targeted analytes. The detection frequencies of florfenicol and florfenicol amine (>60%) were relatively higher in July compared to June and August (Figure 2). Detailed data are listed in Supplemental Table S6. The highest detection frequency of thiamphenicl (51.65%) was observed in August. In the case of ciprofloxacin and oxytetracycline, samples collected in June exhibited the highest detection frequencies. When the temperature rises during summer (from June to September), the growth rate of gastropods also increases and they become more susceptible to bacterial infections [37]. Therefore, the use of antibiotics in farmed gastropods is common during summer to prevent and treat bacterial infections, ultimately reducing high mortality rates and enhancing productivity. 

The highest concentrations of Cd (1.86 mg/kg) and As (7.37 mg/kg) were observed in gastropods collected in September. Interestingly, there was no obvious regularity for levels of Cd and As across different sample months. The accumulation of toxic elements in gastropods can result from various pathways, including water, sediments, or their food sources. This phenomenon is closely tied to human activities, such as industrial pollution, mining, and waste disposal [38,39]. 

Actually, the concentration of antibiotic residues and toxic elements in shrimp can be influenced by a multitude of factors, which can be broadly categorized into environmental, biological, and anthropogenic factors [2]. For example, water quality, temperature, and pH are the main environmental factors. Anthropogenic factors usually include the use of antibiotics, feed composition, and regulatory compliance. 

### 3.4. Health Risk Assessment

Table 4 presents the EDIs of antibiotics, Cd, and As from gastropod consumption in southeast China. The EDIs (Mean, P50, and P95) for florfenicol, oxytetracycline, ciprofloxacin, enrofloxacin, pefloxacin, and metronidazole were all found to be less than 0.1 μg/kg bw/day. Cd EDIs ranged from 0.32 to 0.76 μg/kg bw/day, with the children group having higher Cd exposure than other groups. Regarding As, organic arsenic compounds are generally considered less toxic than inorganic As. Previous reports have shown that inorganic As usually constitutes no more than 5% of the total arsenic in shellfish [40,41]. We calculated the inorganic As by multiplying total As by 5%, and the EDIs of inorganic As ranged between 0.09 and 0.23 μg/kg bw/day. 

**Figure 3:**
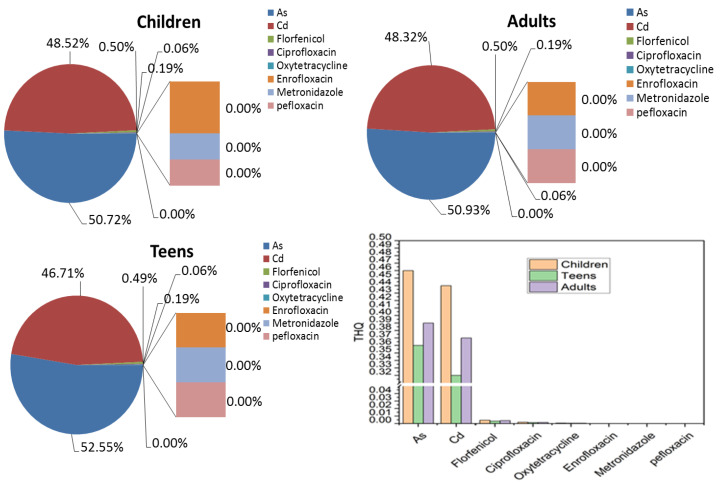
The contribution percentage of THQ for different compounds in gastropods from southeast China.

THQ was used for the health risk assessment by comparing EDIs to ADIs or RfDs. As depicted in Figure 3, THQs of antibiotics, Cd, and As were all less than one, indicating a low health risk. Notably, THQs of Cd and As contributed to over 90% of the total THQs. Other substances, such as florfenicol and ciprofloxacin, did not exceed 5% of the total THQs. This underscores that toxic elements in gastropods from southeast China present a higher health risk than antibiotic residues. The pollution of toxic elements in the marine ecosystem is a global challenge with implications for public health [42,43]. Additionally, we observed higher percentages of Cd and As THQs in the children group than in other groups. The health risks associated with antibiotic residues in gastropods vary among children, teens, and adults due to differences in physiological development and exposure levels. Children, with their immature organ systems and higher metabolic rates, are more susceptible to direct toxic effects and potential disruptions in microbiome development, increasing the risk of allergies and antibiotic resistance [44]. Teens, with their still-developing immune systems, may face similar risks but to a lesser extent, and their behavior might increase exposure [45]. Adults, with fully developed systems, are less vulnerable to direct toxicity but still face long-term risks such as the development of antibiotic-resistant bacteria, especially concerning pregnant women [2]. Overall, while children are at the highest risk, all age groups can be affected, which presents the importance of monitoring and regulating antibiotic residues to protect public health.

**Figure 4:**
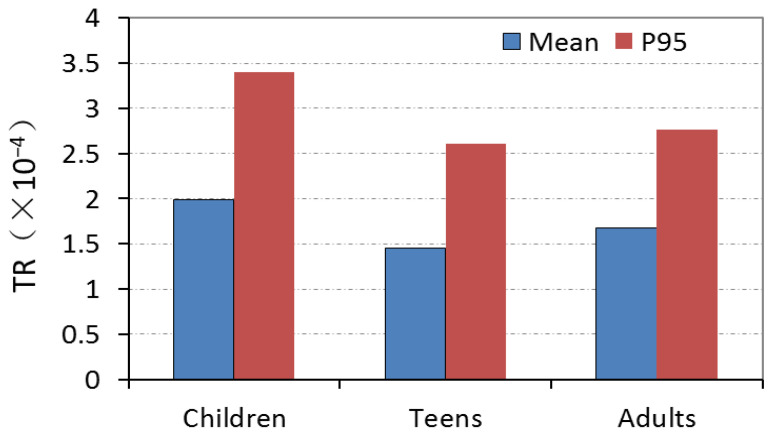
The target cancer risk of As exposure for the consumption of gastropods from southeast China.

Genotoxic and carcinogenic assessments for malachite reen and leucomalachite green employed Margin of Exposure (MOE) based on BMDL_10_ and BMDL_05_. Results showed that MOEs of MG and LMG were greater than 1 × 10^5^, indicating no related health risks from gastropod consumption. Carcinogenic risk assessment for inorganic As, using the cancer slope factor (CSF), revealed lifetime risks of 1 × 10^−4^ to 3.5 × 10^−4^ for children, teens, and adults (Figure 4), suggesting a 1 in 10,000 to 3.5 in 10,000 chance of developing cancer.

## 4. Conclusions

The study investigated antibiotics, malachite green, leucomalachite green, Cd, and As in gastropods from southeast China. Florfenicol, florfenicol amine, and thiamphenicol exhibited high detection frequencies (>35%). Cd and As were widely distributed in gastropod samples, with mean values over 1 mg/kg wet weight. DFs of these chemicals varied across sampling months, peaking for certain substances in June, July, and September. Monte Carlo Simulation (10,000 iterations) revealed EDIs below 1 μg/kg bw per day, except for total As, where organic As compounds dominated (>95%), posing low toxicity. THQs highlighted higher health risks from toxic elements than antibiotics, and leucomalachite green in gastropods. Although dietary exposure indicates low risk, regular monitoring is crucial due to potential marine pollution and increasing gastropod consumption.

## Figures and Tables

**Table 1:** The method validation of organic chemicals in gastropods.

	Antibiotic	Correlation Coefficients (*r*^2^)	Spiking Recovery (*n* = 6)			RSDs (%)	LOQ (μg/kg)
			5 μg/kg	10 μg/kg	50 μg/kg		
QNs	Ciprofloxacin	0.9990	89.5	95.5	96.2	6.7	3.0
	Enrofloxacin	0.9991	90.2	95.6	99.4	7.5	3.0
	Lomefloxacin	0.9985	89.5	96.3	98.8	4.6	3.0
	Norfloxacin	0.9991	92.3	91.9	94.2	5.9	3.0
	Ofloxacin	0.9996	89.5	90.0	91.7	7.7	3.0
	Pefloxacin	0.9997	95.2	97.6	105.9	8.0	3.0
TCs	Chlortetracycline	0.9985	82.8	90.4	94.3	4.9	1.5
	Doxycycline	0.9992	86.2	90.4	92.4	7.2	1.5
	Oxytetracycline	0.9990	86.5	95.5	94.7	8.2	1.5
	Tetracycline	0.9987	81.7	88.8	95.2	7.6	1.5
CAPs	Thiamphenicol	0.9997	90.5	92.4	91.4	5.8	0.1
	Florfenicol	0.9994	94.2	99.8	104.7	7.0	0.2
	Florfenicol Amine	0.9979	84.3	90.1	89.5	7.9	2.0
NMZ	Metronidazole	0.9984	90.2	92.5	101.2	8.1	2.0
Ilegals	Chloramphenicol	0.9995	95.1	99.7	107.5	9.1	0.1
	Malachite green	0.9995	95.6	97.8	103.5	5.6	1.0
	Leucomalachite green	0.9990	94.2	95.0	96.3	9.2	1.0

**Table 2:** The test results of As and Cd in certified reference material (GBW10068 Oyster).

	Certified (mg/kg)	Tested Values (mg/kg)						Mean (mg/kg)	RSD (%)
		Test 1	Test 2	Test 3	Test 4	Test 5	Test 6		
As	12.9 ± 0.6	13.1	12.5	12.8	13.8	12	12.2	12.8	5.2
Cd	18.7 ± 0.7	16.8	17.5	18.5	19.6	18.2	19.9	18.4	6.4

**Table 3:** The distribution of antibiotics, illegal drugs, and toxic elements in gastropods.

	Analytes	*n*	Detection Frequency (%)	Concentration (μg/kg Wet Weight)		MRL (μg/kg)
				Mean	Maximum	
QNs	Enrofloxacin	354	3.67	0.12	12.8	100
	Ciprofloxacin	354	6.21	12.14	1110	100
	Lomefloxacin	354	0.28	0.01	2.55	2
	Norfloxacin	354	0.00	0.00	0	2
	Ofloxacin	354	0.00	0.00	0	2
	Pefloxacin	354	1.41	0.03	4.32	2
TCs	Chlortetracycline	216	0.00	0.00	0	100
	Doxycycline	216	2.78	1.32	0	-
	Oxytetracycline	216	6.02	30.34	3410	200
	Tetracycline	50	0.00	0.00	0	100
CAPs	Thiamphenicol	326	39.88	3.05	136	-
	Florfenicol	287	61.32	46.03	1110	-
	Florfenicol Amine	262	47.33	59.04	2222	-
NMZ	Metronidazole	110	0.91	0.01	0.618	-
Ilegals	Chloramphenicol	336	0.30	0.19	64	prohibit
	Malachite green	113	0.00	0.00	0	prohibit
	Leucomalachite green	113	3.54	0.07	2.48	prohibit
Elements	Cadmiun	303	98.35	1.17	20.8	2.0 (mg/kg)
	Arsenic	304	100.00	6.12	48.2	0.5 (mg/kg)

**Table 4:** Probabilistic results of calculated EDI of antibiotics and toxic elements for children, teens, and adults.

Compounds	ADI	Children (μg/kg bw/Day × 10^−3^)			Teens (μg/kg bw/Day × 10^−3^)			Adults (μg/kg bw/Day × 10^−3^)		
		Mean	P50	P95	Mean	P50	P95	Mean	P50	P95
Florfenicol	10	45.54	45.21	77.99	33.34	33.24	59.86	38.26	38.28	63.14
Oxytetracycline	25	13.72	13.34	23.99	9.8	9.8	18.38	11.52	11.3	19.42
Ciprofloxacin	3	5.26	5.21	9.05	3.85	3.82	6.94	4.42	4.4	7.31
Enrofloxacin	3	0.05	0.05	0.09	0.04	0.04	0.07	0.04	0.04	0.07
Pefloxacin	1.6	0.01	0.01	0.02	0.01	0.01	0.02	0.01	0.01	0.02
Metronidazole	0.6	<0.01	<0.01	0.01	<0.01	<0.01	0.01	<0.01	<0.01	0.01
	**RfD**	**μg/kg bw/day**			**μg/kg bw/day**			**μg/kg bw/day**		
Cd	1	0.44	0.44	0.76	0.32	0.32	0.58	0.37	0.37	0.61
As	(0.3)	2.65	2.64	4.54	1.94	1.94	3.48	2.23	2.23	3.68

Note: The unit of ADI and RfD is μg/kg bw/day. P50 and P95 are the 50th and 95th percentile of the data; the of the data The RfD of inorganic As is 0.3 μg/kg bw/day.

## Data Availability

The original contributions presented in the study are included in the article/supplementary material, further inquiries can be directed to the corresponding author.

## Supplementary Materials

The following supporting information can be downloaded at: https://www.mdpi.com/article/10.3390/foods13081166/s1, Table S1: The mobile phase conditions for antibiotic analysis; Table S2: The mobile phase conditions for illegal drugs and tetrodotoxin; Table S3: The parameters of mass spectrometry for targeted analytes; Table S4: The parameters of ICP-MS for Cd and As; Table S5: Parameters of Monte Carlo simulation for different population in risk assessment; Table S6: The distribution of certain antibiotics in gastropods from different sampling month; Table S7: The distribution of Cd and As in gastropods from different sampling month; Table S8: The formula and structure of analyzed antibiotics.
