# Supplementary material for: Distribution and Probabilistic Risk Assessment of Antibiotics, Illegal Drugs, and Toxic Elements in Gastropods from Southeast China
Source: Foods. 2024 Apr 11;13(8):1166. doi: 10.3390/foods13081166 (PMC11049630; doi:10.3390/foods13081166)
Supplement: Supplementary file 1 [file foods-13-01166-s001.zip › foods-2937626-supplementary.pdf]

Supplementary material

Table S1 The mobile phase conditions for antibiotic analysis

Mobile phase conditions for CAPs (mobile A: 0.05% ammonia solution; B: ACN)		Mobile phase conditions for QNs and TCs (mobile A: 0.1% FA solution; B: MeOH)	
Time (min)	Mobile B (ACN)	Time (min)	Mobile B (MeOH)
0~0.5	10%	0~1.0	10%
0.5~2.0	90%	1.0~4.5	30%
2.0~3.0	90%	4.5~7.0	99%
3.0~3.5	10%	7.0~9.0	99%
3.5~5.0	10%	9.0~9.1	10%

The column is Waters BEH C_{18}（1.7μm，2.1mm×100mm）

Table S2 The mobile phase conditions for illegal drugs and tetrodotoxin

Mobile phase conditions for MG and LMG (mobile A: 5mM ammonium acetate-0.1% formic acid aqueous solution; B: acetonitrile)	
Time (min)	Mobile B (ACN)
0~1	30%
1.0~5.0	95%
5.0~7.0	95%
7.0~7.1	30%
7.1~90	30%

The column for MG and LMG is Waters BEH C18 (1.7μm，2.1mm×100mm)

Table S3 The parameters of mass spectrometry for targeted analytes

Compounds		Molecular formula	Ion source	Precursor Ion (m/z)	Product Ion (m/z)	Collision Energy (eV)	Cone voltage (V)
QNs	Ciprofloxacin	$C_{17}H_{18}FN_3O_3$	ESI+	332.2	314.3/288.3	19/17	36
	Enrofloxacin	$C_{19}H_{22}FN_3O_3$	ESI+	360.3	316.4/342.3	19/23	38
	Lomefloxacin	$C_{17}H_{19}F2N_3O_3$	ESI+	352.3	265.2/308.3	23/17	36
	Norfloxacin	$C_{16}H_{18}FN_3O_3$	ESI+	320.3	302.3/276.3	19/17	50
	Norfloxacin-d_5	$C_{16}H_{13}D_5FN_3O_3$	ESI+	325.1	281.1	12	50
	Ofloxacin	$C_{18}H_{20}FN_3O_4$	ESI+	362.2	318.3/261.2	18/27	38
	Pefloxacin	$C_{17}H_{20}FN_3O_3$	ESI+	334.3	290.3/233.2	17/25	38
TCs	Chlortetracycline	$C_{22}H_{23}ClN_2O_8$	ESI+	479.2	426/444	18/10	40
	Doxycycline	$C_{22}H_{24}N_2O_8$	ESI+	445	428/153.9	14/20	30
	Oxytetracycline	$C_{22}H_{24}N_2O_9$	ESI+	461	426/443	18/10	40
	Tetracycline	$C_{22}H_{24}N_2O_8$	ESI+	445	410/427	25/15	35
	Tetracycline-d_6	$C_{22}H_{18}D_6N_2O_8$	ESI+	445	410	30	35
CAPs	Chloramphenicol	$C_{11}H_{12}C_{l2}N_2O_5$	ESI-	321	152/257	18/16	22
	Chloramphenicol-d_5	$C_{11}H_7D_5Cl_2N_2O_5$	ESI-	326	157	15	25
	Thiamphenicol	$C_{12}H_{15}Cl_2NO_5S$	ESI-	354	185/290	18/10	14
	Florfenicol	$C_{12}H_{14}Cl_2FNO_4S$	ESI-	356	336/185	10/10	25
	Florfenicol amine	$C_{10}H_{14}FNO_3S$	ESI+	248	230/130	10/10	25
NMZ	Metronidazole	$C_6H_9N_3O_3$	ESI+	172.1	128.1/82.1	17/20	45
	Metronidazole-d4	$C_6H_5D_4N_3O_3$	ESI+	176.1	128.1	22	20
Illegal drugs	Malachite green	$C_{23}H_{26}N_2O$	ESI+	329.5	208/313.4	40/35	40
	Malachite green-d_5	$C_{23}H_{21}D_5N_2O$	ESI+	334.4	213.1/318.3	40/40	40
	Leucomalachite green	$C_{23}H_{26}N_2$	ESI+	331.5	239.3/316.4	33/20	40
	Leucomalachite green-d_5	$C_{23}H_{21}D_5N_2$	ESI+	336.4	239.2	30	40

Table S4 The parameters of ICP-MS for Cd and As

Parameter	Value	Parameter	Value
RF power	1550 W	Atomizer	High salt/concentric atomizer
Plasma gas flow rate	15 L/min	Sampling cone/truncation cone	Nickel/Platinum Cone
Carrier gas flow rate	0.80 L/min	Sampling depth	8 mm～10 mm
Auxiliary air flow rate	0.3 L/min	Scan mode	Jumping peak
Helium flow rate	4～5 mL/min	Detection type	automatic
Atomization chamber temperature	2 ℃	points per peak	3
Sample lifting rate	0.3 r/s	Repetitions	3

Table S5 Parameters of Monte Carlo simulation for different population in risk assessment

Variance	Unit	Description	Distribution	Population		
				Children	Teens	Adults
C	mg/kg	Concentration	Log normal	Aalyzed based on different compound		
IR	g/day	Daily consumption	Normal	7.18±3.9	14.69±9.1	20.78±10.5
BW	kg	Body weight	Log normal	16.68±1.48	46.25±1.18	57.03±1.10
Rfd or ADI	mg/kg/day	Reference dose	Fixed	Varied based on different substance		

Table S6 The distribution of certain antibiotics in gastropods from different sampling month.

Sampling month	Enrofloxacin				Florfenicol				Florfenicol Amine			
	n	DF (%)	Mean	Maximum	n	DF (%)	Mean	Maximum	n	DF (%)	Mean	Maximum
5	49	0			29	42.4138	25.33	427	29	24.4828	87.51	2222
6	70	8.6571	0.296	9.13	70	47.1429	20.35	396	52	23.0769	6.2	55.7
7	108	2.7778	0.126	12.8	94	68.0851	49.35	997	87	63.2184	66.16	978
8	98	7.1429	0.089	2.2	68	61.7647	59.81	1110	68	60.2941	78.47	721
9	25	4.00	0.125	3.13	22	53.6364	50.36	165	22	27.2727	16.1	127
Sampling month	Ciprofloxacin				Thiamphenicol				Oxytetracycline			
	n	DF (%)	Mean	Maximum	n	DF (%)	Mean	Maximum	n	DF (%)	Mean	Maximum
5	49	0			29	24.4828	1.19	15.2	29	3.4483	1.562	45.3
6	70	15.7143	38.89	1110	70	28.5714	4.88	136	25	12.00	2.152	42.1
7	108	2.7778	4.07	352	110	52.2727	4.75	97	74	5.4054	0.198	9.9
8	98	8.1633	11.6	434	91	51.6484	4.1	62	62	8.0645	103.86	6410
9	25	0			22	42.0014	1.97	7.59	22	0	0	0

DF (%): detection frequency, the percentage of detected samples to total samples; Concentration unit is μg/kg

Table S7 The distribution of Cd and As in gastropods from different sampling month.

Month	Cd				As			
	n	DF (%)	Mean	Maximum	n	DF (%)	Mean	Maximum
4	30	100	1.41	20.8	30	100	6.00	22.9
5	32	96.88	1.27	12.6	32	100	4.38	12.1
6	112	100	0.99	8.56	114	100	6.65	48.2
7	113	95.58	1.31	12.5	113	100	6.41	29.3
8	8	100	0.05	0.252	8	100	2.17	4.49
9	5	100	1.86	6.99	5	100	7.37	21.8

DF (%): detection frequency, the percentage of detected samples to total samples; Concentration unit of As and Cd is mg/kg.

Table S8 The formula and structure of analyzed antibiotics

Compounds		Chemical formula	Log K_{ow}	Structural formula
QNs	Ciprofloxacin	$C_{17}H_{18}FN_3O_3$	0.28	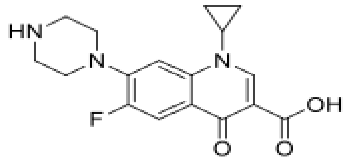
	Enrofloxacin	$C_{19}H_{22}FN_3O_3$	1.1, 2.31	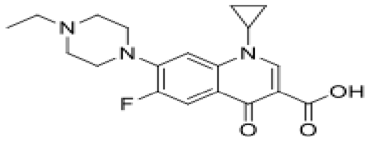
	Lomefloxacin	$C_{17}H_{19}F2N_3O_3$	0.31	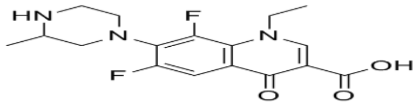
	Norfloxacin	$C_{16}H_{18}FN_3O_3$	-1.03	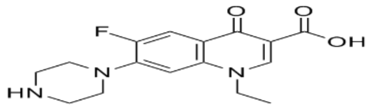

Compounds		Chemical formula	Log K_{ow}	Structural formula
	Ofloxacin	$C_{18}H_{20}FN_3O_4$	-2.0	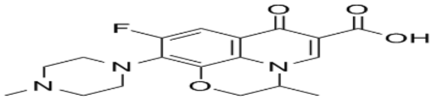
	Pefloxacin	$C_{17}H_{20}FN_3O_3$	-0.27	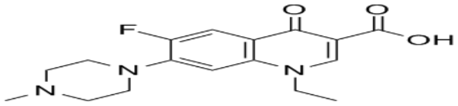
TCs	Chlortetracycline	$C_{22}H_{23}ClN_2O_8$	-0.62	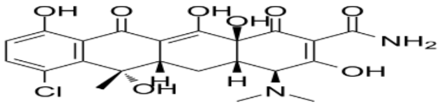
	Doxycycline	$C_{22}H_{24}N_2O_8$	-1.36, -0.02	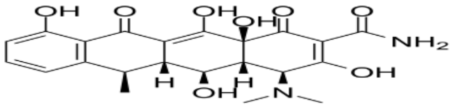
	Oxytetracycline	$C_{22}H_{24}N_2O_9$	-2.87, -0.9	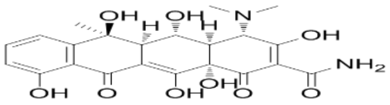

Compounds		Chemical formula	Log K_{ow}	Structural formula
	Tetracycline	$C_{22}H_{24}N_2O_8$	-1.33	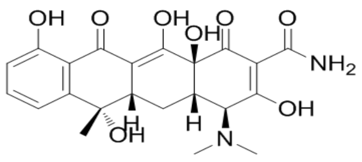
CAPs	Chloramphenicol	$C_{11}H_{12}Cl_2N_2O_5$	0.94, 1.14	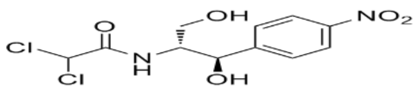
	Thiamphenicol	$C_{12}H_{15}Cl_2NO_5S$	-0.33, -0.27	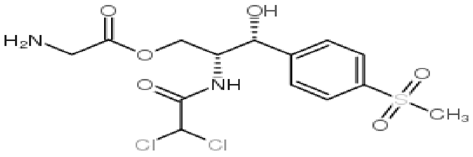
	Florfenicol	$C_{12}H_{14}Cl_2FNO_4S$	-0.04	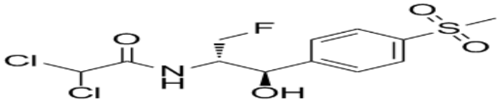
	Florfenicol amine	$C_{10}H_{14}FNO_3S$	-1.27	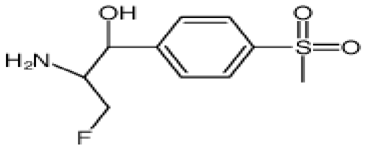

Compounds		Chemical formula	Log K_{ow}	Structural formula
	Metronidazole	$C_6H_9N_3O_3$	-	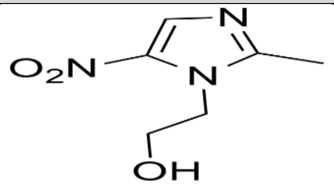
	Malachite green	$C_{23}H_{26}N_2O$	-	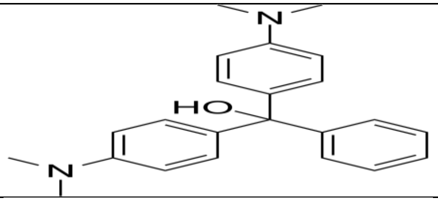
	Leucomalachite green	$C_{23}H_{26}N_2$	-	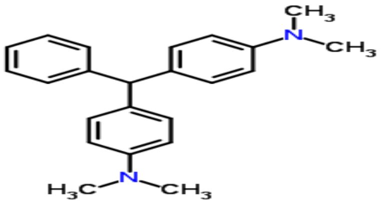
